# Phytochemical Composition and Anti-Inflammatory Activity of Extracts from the Whole-Meal Flour of Italian Durum Wheat Cultivars

**DOI:** 10.3390/ijms16023512

**Published:** 2015-02-04

**Authors:** Barbara Laddomada, Miriana Durante, Fiorenza Minervini, Antonella Garbetta, Angela Cardinali, Isabella D’Antuono, Sofia Caretto, Antonio Blanco, Giovanni Mita

**Affiliations:** 1 Institute of Sciences of Food Production, CNR, 73100 Lecce, Italy; E-Mails: miriana.durante@ispa.cnr.it (M.D.); sofia.caretto@ispa.cnr.it (S.C.); giovanni.mita@ispa.cnr.it (G.M.); 2 Institute of Sciences of Food Production, CNR, 70125 Bari, Italy; E-Mails: fiorenza.minervini@ispa.cnr.it (F.M.); antonella.garbetta@ispa.cnr.it (A.G.); angela.cardinali@ispa.cnr.it (A.C.); isabella.dantuono@ispa.cnr.it (I.D.); 3 Department of Soil, Plant and Food Sciences, Section of Genetic and Plant Breeding, University of Bari Aldo Moro, 70124 Bari, Italy; E-Mail: antonio.blanco@agr.uniba.it

**Keywords:** durum wheat, bioactive compounds, phenolic acids, isoprenoids, interleukin 8, transforming growth factor β1

## Abstract

In this study, the quali-quantitative composition of hydrophilic (phenolic acids) and lipophilic (isoprenoids) extracts from whole-meal flour of five elite Italian durum wheat cultivars was determined. Significant differences in the content of bioactive compounds were observed among the wheat extracts, in particular concerning the content of bound phenolic acids, lutein and β-tocotrienols. The cultivars Duilio and Svevo showed the highest amount of phenolic acids and isoprenoids, respectively. Extracts were evaluated for their anti-inflammatory activity on HT-29 human colon cells by measuring the levels of interleukin 8 (IL-8) and transforming growth factor β1 (TGF-β1). Durum wheat extracts significantly inhibited the secretion of the pro-inflammatory IL-8 mediator at 66 µg/mL of phenolic acids and at 0.2 µg/mL of isoprenoids. Conversely, the secretion of the anti-inflammatory mediator TGF-β1 was not modified by neither hydrophilic nor lipophilic extracts. These results provide further insight into the potential of durum wheat on human health suggesting the significance of varieties with elevated contents of bioactive components.

## 1. Introduction

Durum wheat (*Triticum turgidum* ssp. *durum* (Desf.) Husnot) is the most cultivated tetraploid species (2*n* = 28, genomes AABB) and economically important cereal in the Mediterranean area, because it is particularly adapted to semiarid areas and represents the preferred raw material for making pasta, couscous and different types of bread.

The interest in durum wheat goes beyond nutritional requirements. In fact, dietary intake of whole wheat products has been associated with a reduced risk for developing metabolic diseases [1]. The health benefits of whole wheat food products depend on a range of dietary fibre, mineral components and phytochemicals, including phenolic compounds, carotenoids and tocols, which are concentrated mostly in the bran and germ portions of the kernel [2,3]. The phytochemical composition of wheat species and varieties has been extensively described [4], and the enduring attention to wheat bioactive compounds is documented by a number of genetic studies to assess their heritability [5,6,7].

This information, together with ongoing genome sequencing and gene annotation work, are providing a wealth of knowledge for the potential improvement of the content of healthy compounds in the kernel of cultivated wheat [8,9,10]. Nevertheless, the precise beneficial effects of wheat phytochemicals are still under debate because of the great number of potential health-promoting compounds and the complexity of studying their biological effects [1].

Phenolic acids are the most abundant wheat phytochemicals with health-related antioxidant activity [11]. The majority of phenolic acids are insoluble, because they are covalently cross-linked with cell wall polymers [12]. Although considered poorly bioavailable, the bound forms of phenolic acids can reach the colon where they exert antioxidant activity and contribute to a reduced risk for colorectal cancer [13]. Durum wheat grain is also a moderate source of isoprenoids, such as tocols and carotenoids, and contains higher amounts of these components compared to other wheat species, probably due to breeding programs for a bright yellow color [5,14].

Some evidence suggests that the consumption of whole wheat food products provides unique bioactive compounds that complement those present in fresh fruits and vegetables [15]. In fact, wheat phytochemicals may impact the oxidative status of cells, thus preventing tissue injury and inflammation [16,17].

Among inflammatory mediators, cytokines act in local and intercellular communications. One cytokine implicated in chronic inflammation is interleukin 8 (IL-8) [18], a member of the chemokine family that initially was identified in a number of inflammatory diseases involving neutrophil activation [19]. Aberrant IL-8 production results in chronic inflammatory conditions [20,21,22], such as inflammatory bowel disease (IBD), an important inflammatory disease that affects humans worldwide [23,24,25]. IL-8 inhibition, could lead to a reduction of neutrophil accumulation into the inflammatory sites [26].

Another cytokine, TGF-β1, produced by the HT-29 intestinal cell line, contributes in regulating the intestinal barrier function by acting on epithelial tight junctions and exhibiting anti-inflammatory activity against commensal bacteria [27,28]. In addition, TGF-β1 is a potent enhancer of epithelial cell restitution, a process by which epithelial continuity is rapidly re-established after various forms of injury [29]. Along with the B cell activating factor of the tumor necrosis factor (TNF) family and a proliferation-induced ligand, TGF-β produced by intestinal epithelial cells is able to promote immunoglobulin A (IgA) switching [30].

Recent studies were conducted to investigate the anti-inflammatory activity of ethanolic extracts from whole wheat-bread flour [31]. Nevertheless, to date, no reports are available about the effect of phenolic acids (including the most abundant bound forms) and isoprenoids of whole-meal flour from durum wheat on the secretion of IL-8 and TGF-β1 inflammatory mediators.

Our aims were (i) to determine the quali-quantitative composition of hydrophilic (phenolic acids) and lipophilic (isoprenoids) extracts from whole-meal flour of five Italian durum wheat elite cultivars; and (ii) to evaluate the anti-inflammatory activity of the extracts by measuring IL-8 and TGF-β1 levels on the lipopolysaccharide (LPS)-stimulated HT-29 human intestinal cell line.

## 2. Results and Discussion

### 2.1. Composition of Phenolic Extracts

Phytochemical analyses were carried out on the hydrophilic and lipophilic extracts from the whole-meal flour of five durum wheat cultivars (Ciccio, Duilio, Svevo, Iride and Aureo) selected among the foremost Italian elite varieties.

The hydrophilic extracts were analyzed for their content in individual bound, conjugated and free phenolic acids (Table 1). The overall results indicated that ferulic acid was the most abundant phenolic acid among the five cultivars, particularly as a bound insoluble form. In fact, bound ferulic acid represented about 78% of total phenolic acids and varied from a minimum of 302.09 μg/g dry matter (dm) to a maximum of 431.67 μg/g dm, with Duilio and Ciccio showing the highest contents (Table 1). Besides ferulic acid, other bound phenolic acids were detected, namely *p*-coumaric acid, vanillic acid, syringic acid and 4-hydroxybenzoic acid. In particular, *p*-coumaric acid was the second-most represented bound phenolic acid (ranging from 5.88 μg/g dm in Svevo and 11.25 μg/g dm in Duilio), followed by vanillic, syringic and 4-hydroxybenzoic acids.

Sinapic acid was the prevalent conjugated phenolic acid and it was followed for abundance by the conjugated ferulic, vanillic, syringic, 4-hydroxybenzoic and *p*-coumaric acids. Finally, among the soluble free phenolic acids, ferulic acid was again the most represented (varying from 1.41 to 1.69 μg/g dm).

**Table 1:** Composition and content of individual bound, conjugated and free phenolic acids (µg/g dry matter) in the whole meal flour of five durum wheat, Italian, elite cultivars.

Phenolic Acids	Durum Wheat Cultivars				
	Ciccio Mean ± SD μg/g dm	Duilio Mean ± SD μg/g dm	Svevo Mean ± SD μg/g dm	Iride Mean ± SD μg/g dm	Aureo Mean ± SD μg/g dm
**Ferulic acid**					
Bound	408.54 ^a,b^ ± 29.6	431.67 ^a^ ± 17.7	333.60 ^c^ ± 27.8	345.61 ^b,c^ ± 23.2	302.09 ^c^ ± 25.8
Conjugated	23.21 ^a,b^ ± 0.86	26.32 ^a,b,c^ ± 1.65	28.92 ^c,d^ ± 1.31	22.71 ^a^ ± 1.12	27.55 ^b,d^ ± 2.07
Free	1.41 ^a^ ± 0.03	1.62 ^a^ ± 0.09	1.57 ^a^ ± 0.07	1.60 ^a^ ± 0.26	1.69 ^a^ ± 0.03
***p*-Coumaric acid**						
Bound	8.72 ^a^ ± 0.42	11.25 ^b^ ± 0.79	5.88 ^c^ ± 0.69	7.92 ^a,c^ ± 1.14	8.47 ^a^ ± 0.83	
Conjugated	0.85 ^a,b^ ± 0.09	0.62 ^b,c^ ± 0.09	0.44 ^c^ ± 0.10	0.51 ^c^ ± 0.10	1.05 ^a^ ± 0.19	
Free	0.87 ^a^ ± 0.08	0.85 ^a^ ± 0.03	0.76 ^a,b^ ± 0.04	0.70 ^b^ ± 0.02	0.80 ^a,b^ ± 0.03	
**Vanillic acid**						
Bound	5.02 ^a,b^ ± 0.13	5.21 ^a^ ± 0.53	3.85 ^b,c^ ± 0.46	3.24 ^c^ ± 0.56	4.55 ^a,b^ ± 0.56	
Conjugated	4.44 ^a^ ± 0.09	5.79 ^b^ ± 0.33	5.08 ^c^ ± 0.10	4.66 ^a,c^ ± 0.09	5.80 ^b^ ± 0.08	
Free	1.43 ^a,b^ ± 0.03	1.62 ^c^ ± 0.09	1.36 ^a^ ± 0.05	1.36 ^a^ ± 0.04	1.58 ^c,b^ ± 0.06	
**Syringic acid**						
Bound	4.71 ^a^ ± 0.58	3.87 ^a,b^ ± 0.13	3.04 ^b,c^ ± 0.12	2.71 ^c^ ± 0.38	1.19 ^d^ ± 0.08	
Conjugated	1.78 ^a,b,c^ ± 0.18	2.23 ^a,b^ ± 0.51	2.45 ^a^ ± 0.27	1.64 ^b,d^ ± 0.19	1.11 ^c,d^ ± 0.17	
Free	<0.01	<0.01	<0.01	<0.01	<0.01	
**4-Hydroxibenzoic acid**						
Bound	0.61 ^a^ ± 0.03	0.87 ^b^ ± 0.02	0.87 ^b^ ± 0.04	0.71 ^c^ ± 0.02	0.37 ^d^ ± 0.02	
Conjugated	1.23 ^a^ ± 0.02	1.75 ^b^ ± 0.09	2.67 ^c^ ± 0.04	3.21 ^d^ ± 0.02	1.98 ^e^ ± 0.02	
Free	<0.01	<0.01	<0.01	<0.01	<0.01	
**Sinapic acid**						
Bound	<0.01	<0.01	<0.01	<0.01	<0.01	
Conjugated	79.12 ^a,b^ ± 2.22	74.09 ^a^ ± 5.94	86.38 ^b^ ± 1.63	76.83 ^a,b^ ± 3.30	107.39 ^c^ ± 4.74	
Free	<0.01	<0.01	<0.01	<0.01	<0.01	

Samples with same letter within each row are not significantly different (*n* = 3, *p* < 0.05).

**Figure 1:**
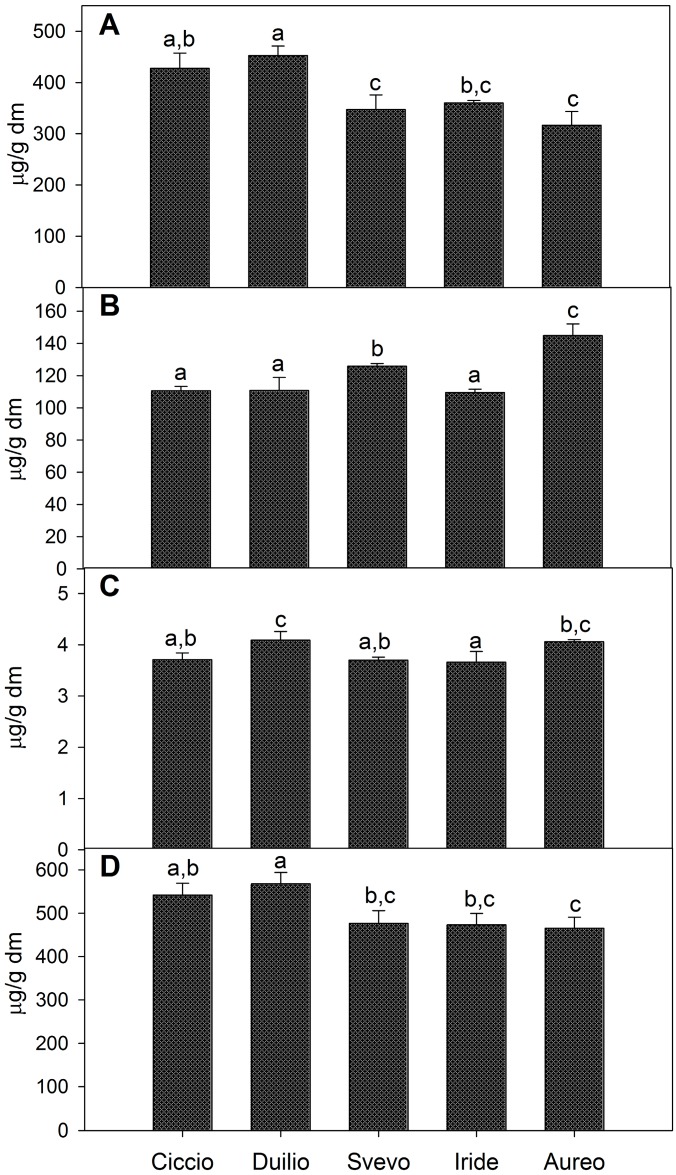
Content of total bound (**A**); total conjugated (**B**); total free phenolic acids (**C**); and total phenolic acids (**D**) as the sum of bound, conjugated and free phenolic acids, expressed as μg/g dm, in the whole meal flour of five, Italian, durum wheat cultivars. Different letters within each panel indicate significant differences between samples (*n* = 3, *p* < 0.05).

The variations for total bound, total conjugated and total free phenolic acids observed among the five whole-meal flour extracts are shown in Figure 1. In particular, Duilio showed the highest content of total bound forms (453 μg/g dm), which was significantly different from that of Svevo, Iride and Aureo. The lowest content of bound phenolic acids was observed in Aureo (317 μg/g dm) (Figure 1A). The conjugated phenolic acids also showed significant variations among the samples with 110 μg/g dm in Ciccio, Duilio and Iride and a higher content in Aureo (145 μg/g dm) (Figure 1B). Total free phenolic acids were less abundant compared with the other two forms and were fairly different among the cultivars (Figure 1C). The sum of total bound, total conjugated and total free phenolic acids is reported (Figure 1D), confirming that the highest content of total phenolic acids was found in the Duilio extract (567.75 μg/g dm), followed by Ciccio (541.97 μg/g dm), Svevo (476.87 μg/g dm), Iride (473.41 μg/g dm) and Aureo (465.60 μg/g dm).

In general, bound phenolic acids accounted for about 75.4% of total phenolic acids, whereas the soluble conjugated and free forms contributed for ~24% and 1%, respectively. The present results confirm previous findings and suggest that modern wheat varieties have similar or even higher amounts of phenolic acids compared to old varieties and wild ancestors [32,33].

Previous studies on durum wheat Italian varieties revealed no significant differences for phenolic acid content, as determined by the Folin–Ciocalteu procedure. Nevertheless, in agreement with our findings, it has been reported that the high-performance liquid chromatography-electrospray time-of-flight mass spectrometry (HPLC–ESI-TOF-MS) analysis highlighted remarkable differences among the genotypes, thus confirming HPLC as the best methodology to analyze phenolic content in whole wheat meal flour [34].

As extensively documented, phenolic acid content in wheat may be largely influenced by a number of environmental factors. In fact, significant correlations between phenolic acid content and environmental factors were observed in wheat and even highly heritable phenolic acid components may differ in amount over different years and sites [32,33]. Further evidence suggests that it is possible to produce whole durum wheat grain rich in phenolic acids by selecting the most suitable location and adopting eligible cultivars [35]. In a three-year study at Valenzano (Bari, Italy) on 112 tetraploid wheat genotypes, we found that cultivars Ciccio, Svevo, Duilio, Iride and Aureo were quite stable over the years, showing only slight variations in phenolic acid content (unpublished data).

### 2.2. Composition of Isoprenoid Extracts

**Figure 2:**
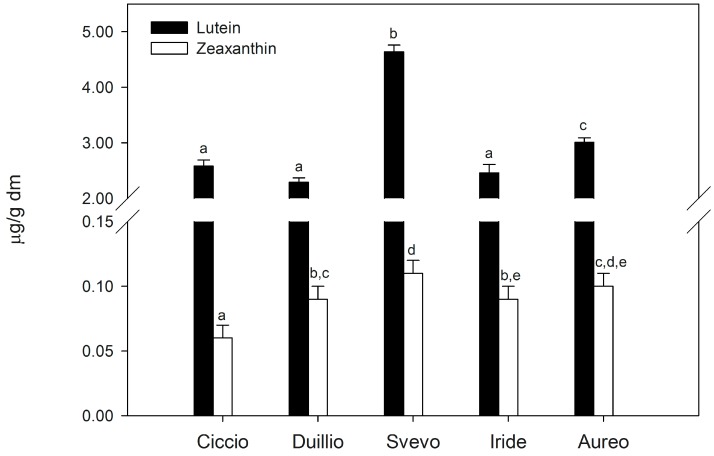
Content of carotenoids, expressed as μg/g dm, in the whole meal flour of five, Italian, durum wheat cultivars. Different letters, within each histogram series, indicate significant differences between different samples (*n* = 3, *p* < 0.05).

The characterization of isoprenoid extracts by HPLC analysis enabled an accurate identification and quantification of carotenoids and tocols. Figure 2 indicates that lutein was the most abundant carotenoid in all samples, confirming previous studies about primitive and modern wheat species [36]. As showed in Figure 2, the whole-meal flour extract from Svevo showed the highest lutein content (4.64 μg/g dm)*.* Among carotenoids, zeaxanthin was also detected, varying from 0.06 μg/g dm, in Ciccio, to 0.11 μg/g dm, in Svevo (Figure 2). Other carotenoids, such as α- and β-carotene, were found only in trace amounts (data not shown). These results are in agreement with those reported by Lachman* et al.* [13] showing that the most abundant carotenoid in the grains of various wheat species is lutein (~83%), followed by zeaxanthin (~10%) and β-carotene (~7%).

**Figure 3:**
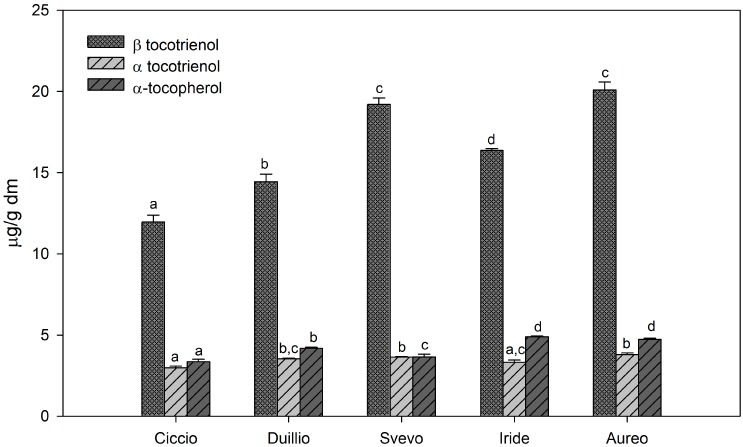
Content of tocols, expressed as μg/g dm, in the whole meal flour of five, Italian, durum wheat cultivars. Different letters, within each histogram series, indicate significant differences between different samples (*n* = 3, *p* < 0.05).

Figure 3 shows the content of individual tocols detected among the considered isoprenoid extracts. Indeed, β-tocotrienol was identified as the main tocol in all samples (on average ~16.41 μg/g dm), followed by α-tocopherol and α-tocotrienol (on average ~4.16 and 3.46 μg/g dm, respectively). The amount of β-tocotrienol was significantly higher in Aureo and Svevo extracts (20.09 and 19.21 μg/g dm, respectively) compared to other samples. Conversely, α-tocopherol and α-tocotrienol, besides being less represented, showed slight variations among the samples (Figure 3).

Overall, our results showed that the whole-wheat flour from different durum wheat cultivars may vary significantly in tocol and carotenoid content. According to the results obtained, the content in total carotenoids and tocols varied from 31 μg/g dm (Svevo) to 21 μg/g dm (Ciccio).

### 2.3. Effect of Hydrophilic and Lipophilic Extracts on IL-8 and TGF-β1 Production by HT-29 Cells

The hydrophilic extract from Duilio showed the highest content of total phenolic acids (Table 1, Figure 1) while the Svevo lipophilic extract, exhibited the highest content of isoprenoids (Figure 2 and Figure 3).

**Figure 4:**
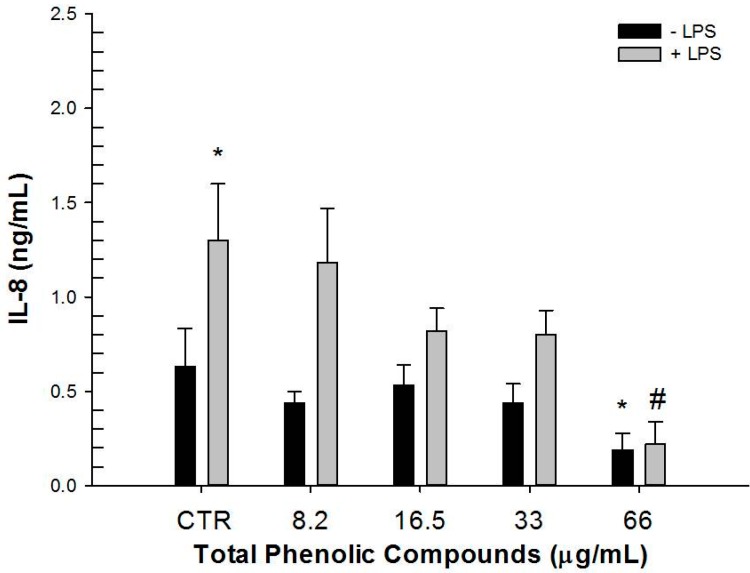
Basal (−LPS) and stimulated (+LPS) IL-8 secretion levels produced by HT-29 human intestinal cell line treated with different phenolic acids concentrations from Duilio extract as compared to vehicle control (CTR). * *p* < 0.05* vs.* CTR − LPS; # *p* < 0.05* vs.* CTR + LPS (*n* = 12).

Hydrophilic and lipophilic extracts from Duilio and Svevo were also assayed for their antioxidant activity. The hydrophilic extracts showed 86.81 and 77.34 µmol Trolox equivalents (TE) dm; while lipophilic extracts revealed 14.49 and 15.83 µmol TE dm (Duilio and Svevo, respectively). Different amounts of Duilio and Svevo extracts were then tested on the LPS-stimulated HT-29 human intestinal cell line to measure the secretion of IL-8 and TGF-β1 inflammatory mediators (Figure 4). In fact, HT-29 human colon cells respond to inflammatory stimuli and bacterial invasion in a similar manner to intestinal epithelial cells, by expressing chemokine receptor analogues and showing similar immune regulatory mechanisms [37,38].

Preliminary experiments were carried out to select the maximum non-toxic concentration of both extracts by using the MTT test on the HT-29 cell line after 24 h of exposure (data not shown). In addition, the effect of methanol and DMSO, used as vehicles to solubilize the hydrophilic and lipophilic extracts, on cytokine secretion was tested. As a result, in agreement with previous evidence, DMSO reduced IL-8 secretion [39,40,41]. Also, in our experimental conditions, an analogous effect was observed for methanol. Thus, to take into account these results, vehicle treated samples were used as controls (Figure 4).

Results showed that in stimulated cells (+LPS), the hydrophilic extract determined a dose dependent reduction of IL-8 secretion with a significant effect at 66 µg/mL total phenolic acids (66 µg phenolic acids were extracted from 110 mg whole meal flour). Likewise, this concentration of total phenolic acids significantly reduced the basal (−LPS) IL-8 secretion compared to vehicle control sample. A significant reduction of IL-8 secretion was only observed in stimulated cells (+LPS) when lipophilic extract at 0.2 µg/mL total isoprenoids (0.2 µg isoprenoids extracted from 6 mg whole meal flour) was used (Table 2).

As expected, extracts from cultivars containing lower amounts of phenolic acids and isoprenoids did not determine any reduction of IL-8 secretion with or without LPS-stimulation (data not shown).

Concerning TGF-β1, under our experimental conditions, neither LPS stimulation nor vehicles influenced its secretion (data not shown)*.* Moreover, the exposure of HT-29 cells to either hydrophilic or lipophilic extracts did not modify the TGF-β1 levels at any of the tested concentrations when compared to vehicle controls.

As a result, the activity of Duilio hydrophilic extract most probably was cytokine-related, because no significant TGF-β1 modification was observed in any of the tested experimental conditions.

The results presented here show a synergistic effect of the bioactive compounds present either in hydrophilic or lipophilic whole-meal flour extracts in terms of anti-inflammatory activity, rather than anti-inflammatory effects of individual compounds. Whent* et al.* [31] observed a correlation between particular wheat phenolic acids and isoprenoids and other inflammation mediators (IL-1β and TNF-α) in mouse macrophages. Nevertheless, the same authors remarked that a definite conclusion about the role of individual compounds could not be drawn. Moreover, due to the large number of bioactive components contained in wheat grains, it would be difficult to assign the potential anti-inflammatory activity entirely to individual components [31].

The* in vitro* results presented here show the potential anti-inflammatory activity of phenolic acids from durum wheat whole-meal flour, confirming previous results on bread wheat extracts and bread wheat food products [31,42]. Moreover, our results provide novel indications about the activity of phenolic compounds on human colon cells, rather than on macrophages and aortic endothelial cells, as previously investigated [43].

The results obtained appear to confirm that a significant reduction of pro-inflammatory cytokines in humans can occur after the consumption of bioprocessed bread, with improved bioavailability of phenolic compounds [42]. More recently, it has been shown that most dietary phenolic acids are not metabolized in the stomach and small intestine, but reach the colon intact where they are released by the local microflora and are allowed to exert antioxidant and anti-inflammatory activity locally [12,44]. On the other hand, the amounts of phenolic acids that were effective in this study could be easily found in the colon after the consumption of a common wheat serving (75 g) containing 5–7 mg phenolic acids [45]. Moreover, the use of whole grains in cereal products can further increase the phenolic acid intake. However, bioavalability studies measuring polyphenol concentrations in plasma and tissues can definitely assess the healthy effects for humans [45,46].

Concerning lipophilic extract from durum wheat, to the best of our knowledge, this is the first report on the anti-inflammatory activity observed as a dose-dependent reduction in IL-8 concentrations in LPS-stimulated HT-29 cells. Finally, other* in vitro* studies that considered different cellular models and inflammatory mediators demonstrated anti-inflammatory activity of individual lipophilic components, though at higher concentrations than those tested in the present study [47].

**Table 2:** Basal (−LPS) and stimulated (+LPS) IL-8 secretion levels produced by HT-29 human intestinal cell line treated with different isoprenoids concentrations from Svevo extract. Different letters indicate significant differences between compounds (*n* = 12, *p* < 0.05). The comparison is between isoprenoids treatments* vs.* vehicle control (CTR) ± LPS (*n* = 12, * *p* < 0.05).

IL-8 (ng/mL)		
SVEVO Total Isoprenoids (μg/mL)	−LPS	+LPS
CTR	0.33 ± 0.02 ^a^	1.77 ± 0.17 ^b^
0.01	0.37 ± 0.09	1.42 ± 0.27
0.02	0.36 ± 0.06	1.80 ± 0.40
0.1	0.31 ± 0.07	1.88 ± 0.23
0.2	0.34 ± 0.04	1.34 ± 0.33 *

## 3. Experimental Section

### 3.1. Wheat Samples

Five elite Italian durum wheat cultivars, namely Ciccio, Svevo and Iride (released in 1996); Duilio (released in 1984); and Aureo (released in 2009), were evaluated for the content and composition of phenolic acids and isoprenoids. The plant material was grown under conventional farming in the experimental field of the University of Bari, at Valenzano (Bari, Italy) in the 2009-10 growing season. The cultivars were grown according to a randomized complete block design (RCB) to take into account any environmental effect on phytochemical accumulation in the grain. The software MSTATC, function EXSERIE, was used as experiment design program [48]. The RCB comprised three replications and plots consisting of 1 m rows, 30 cm apart, with 50 germinating seeds per plot. Preliminary analysis of bioactive components in the whole-meal flour of the cultivar Ciccio showed the lack of significant differences among the plots, thus three grams of whole grain from each experimental replication, corresponding to each cultivar, were pooled into one sample that was subsequently used for further analyses. The whole grain samples were milled to a fine powder, immediately cooled to −20 °C and kept at this temperature until analysis to protect bioactive components from degradation.

### 3.2. Extraction of Phenolic Acids from Whole-Meal Flour and HPLC Analysis

Phenolic acids present in the whole wheat grain exist as soluble free acids, soluble conjugates (that are esterified to sugars and other low-molecular-mass molecules), and as insoluble bound form linked to cell wall structural components such as cellulose, lignin and proteins through ester bonds. Phenolic acids (soluble free, soluble conjugated and bound), were extracted from the whole-meal flour samples according to Li* et al.* [11]. Briefly, free phenolic acids were extracted using an 80/20 ethanol/water (*v*/*v*) solvent mixture. Conjugated phenolic acids were released after alkaline hydrolysis of this initial extract (2 M NaOH, 4 h), and insoluble bound phenolic acids were released via alkaline hydrolysis (2 M NaOH, 4 h) of the residue from the initial ethanol/water extraction. Both conjugated and bound phenolic acid fractions were acidified to pH 2 (12 M HCl) after hydrolysis to enable extraction into organic solvent. The extracted compounds were analysed by HPLC using an Agilent 1100 high-performance liquid chromatography equipped with a photodiode array detector (DAD), (Agilent Tecnologies, Waldbronn, Germany). The wavelengths used for quantification of phenolic acids were 280, 295 and 320 nm. Phenolic acid separation was achieved according to Li* et al.* [11] with some modifications by using a Phenomenex-luna 5 μm C18 (2) 100 Å column (250 × 4.6 mm), (Phenomenex, Torrance, CA, USA) and the temperature of the column was set at 30 °C. The flow rate of the mobile phase was 1.0 mL/min, and the injection volume was 20 μL. A gradient elution program was utilized with a mobile phase consisting of acetonitrile (solution A) and 1% (*v*/*v*) H_3_PO_4_ in water (solution B) as follows: Isocratic elution, 100% B, 0–30 min; linear gradient from 100% B to 85% B, 30–55 min; linear gradient from 85% B to 50% B, 55–80 min; linear gradient from 50% B to 30% B, 80–82 min; post time, 10 min before the next injection. All phenolic acids were quantified *via* rationing to the internal standard (3,5-dichloro-4-hydroxybenzoic acid) added to each sample and using calibration curves of phenolic acid standards having undergone the same extraction procedure.

### 3.3. Extraction of Isoprenoids from Whole-Meal Flour and HPLC Analysis

Isoprenoids (tocols and carotenoids) were extracted by mild saponification as described by Panfili* et al.* [49] with slight modifications. Briefly, 0.5 g sample was incubated in a screw-capped tube, covered with aluminum foil, with 2 mL methanolic KOH (60%, *w*/*v*), 2 mL ethanol (20% *v*/*v*), 1 mL NaCl (0.1% *w*/*v*) and 5 mL BHT (0.05% *w*/*v*) in acetone. After alkaline digestion at 60 °C for 30 min and subsequent cooling, 15 mL 1% (*w*/*v*) sodium chloride solution was added. The sample was then extracted twice with 15 mL *n*-hexane/ethyl acetate (9/1, *v*/*v*). The upper phase, containing the isoprenoids, was collected into amber glassware and dried under nitrogen flux. The dried material was redissolved in 1 mL ethyl acetate, filtered through a 0.45 μm syringe filter (Millipore Corporation, Billerica, MA, USA) and assayed by HPLC. Separation was carried out using an Agilent 1100 Series HPLC system (Agilent Tecnologies, Waldbronn, Germany) as described by Fraser* et al.* [50] with slight modifications. Isoprenoids were separated using a reverse-phase C30 column (5 μm, 250 × 4.6 mm) (YMC Inc., Wilmington, NC, USA) with mobile phases consisting of methanol (A), 0.2% ammonium acetate aqueous solution/methanol (20/80 *v*/*v*) (B), and *tert*-methyl butyl ether (C). The isocratic elution was as follows: 0 min, 95% A and 5% B; 0 to 12 min, 80% A, 5% B, and 15% C; 12 to 42 min, 30% A, 5% B, and 65% C; 42 to 60 min, 30% A, 5% B, and 65% C; 60 to 62 min, 95% A, and 5% B. The column was re-equilibrated for 10 min between runs. The flow rate was 1.0 mL/min and the column temperature was maintained at 25 °C. The injection volume was 10 μL. Absorbance was registered by DAD at wavelengths of 475 nm for carotenoids and 290 nm for tocols. Isoprenoids were identified by comparing their retention times and UV-vis spectra to authentic standards.

### 3.4. Determination of the Trolox Equivalent Antioxidant Capacity (TEAC)

Antioxidant activity of hydrophilic and lipophilic extracts were analyzed in the phenolic acid and isoprenoid extracts of Duilio and Svevo by the TEAC assay as described by Re* et al.* [51] using the radical cation ABTS•+ and Trolox as standard. Whole-meal flour (0.5 g) was subjected to hydrophilic and lipophilic extraction, the extracts were dried under nitrogen flux and suspended in 1 mL of 5 mM phosphate buffered saline (PBS), pH 7.4 water and ethanol respectively. Ten microliters of each extract were assayed into 1 mL of the reaction mixture and the absorbance decrease was measured at 734 nm. PBS and ethanol were used as control for hydrophilic and lipophilic extracts respectively. A Trolox calibration curve in a range of 2.5–20 μM was prepared under the same conditions of the samples. The antioxidant capacity of the samples was calculated, on the basis of the inhibition exerted by standard Trolox concentrations at 734 nm, inhibition time being fixed at 10 min. Results were expressed as µmol of TE per gram of sample.

### 3.5. Quantification of IL-8 and TGF-β Produced by HT-29 Cell Line with and without LPS Stimulation

The hydrophilic and lipophilic extracts were dried under nitrogen flux and suspended in methanol or DMSO, respectively, and used on HT-29 human cells to evaluate IL-8 and TGF-β1 secretion. HT-29 cells (ECACC, Sigma Aldrich, St. Louis, MO, USA) were grown in 25 cm^2^ flasks at a starting density of 250,000 cells/mL in a McCoy 5A medium with 3 g/L glucose, supplemented with 10% of foetal bovine serum, 1% of l-glutamine, 1% of antibiotic and antimycotic solution. The cells were harvested twice a week up to 70%–80% confluence using a Trypsin-EDTA solution. Cell density and viability were determined by Scepter automated cell counter (Millipore, Milan, Italy). The cells used for experimental protocols showed a mean viability of 90%. Subsequently, dose-dependent experiments were performed in 24-well plates seeding 900 µL per well of HT-29 cellular suspension (at density of 1 × 10^6^ cells). After overnight incubation, cells were exposed to 100 µL of different concentrations of phenolic or isoprenoid extracts, incubated for further 24 h in combination with or without lipopolysaccharide (LPS, 1 µg/mL). The plates were centrifuged and the cell culture supernatants were collected and tested with ELISA Kits (BD OptEIA set human IL-8 and TGF-β1)*.* The production of IL-8 and TGF-β1 were measured according to the manufacturer’s instructions. The concentration of IL-8 in the samples was assessed by a standard curve (ranging from 0.08 to 5 ng/mL) obtained by linear regression analysis of purified IL-8 concentration* versus* the optical density in a double logarithmic plot. The concentration of TGF-β1 in the samples was assessed by a standard curve ranging from 0.5 to 32 ng/mL. Preliminary experiments were carried out in 96-well plates, following the protocol described by Minervini* et al.* [52] in order to assess the highest non-cytotoxic concentration of phenolic and isoprenoid extracts by 3-(4,5-dimethylthiazol-2-yl)-2,5-diphenyltetrazolium bromide) tetrazolium reduction assay (MTT) after 24 h of exposure.

### 3.6. Statistical Analysis

Data were analyzed using SigmaStat version 11.0 software (Systat Software Inc., Chicago, IL, USA). Chemical data are presented as mean values ± standard deviation of three independent experiments. One-way ANOVA followed by Bonferroni’s *post-hoc* comparisons tests was performed to establish significant differences between means (*p* < 0.05). Data referring to cytokine analyses are presented as mean value ± standard deviation of two independent experiments (*n* = 12). A *t*-test was used to assess the differences in IL-8 and TGF-β1 concentrations between control samples. One-way ANOVA, followed by Dunn’s method, was used to compare the controls and treated samples. Values with *p* < 0.05 were considered significantly different.

## 4. Conclusions

Wheat is an important component of the human diet, and the effect on human health of bioactive compounds present in the germ and bran portions of wheat has becoming a fascinating and important subject of research. The beneficial effects of wheat are still under debate because of the great number of potential health-promoting components present in the grains and the complexity of studying their biological effects.

This work provides novel insights into the quali-quantitative composition of durum wheat bioactive metabolites in relation to their potential health-promoting activity. The data show that phenolic acids and isoprenoids from whole meal extracts inhibited the secretion of the pro-inflammatory IL-8 mediator. On the contrary, in our experimental conditions, the exposure of HT-29 cells to both hydrophilic and lipophilic extracts did not modify the concentrations of the anti-inflammatory TGF-β1 mediator.

## Notes

Mention of trade names of wheat cultivars is solely to provide specific information and not to imply any recommendation or endorsement.

## References

[1] Fardet A. (2010). New hypotheses for the health-protective mechanisms of whole-grain cereals: What is beyond fibre?. Nutr. Res. Rev..

[2] Durante M., Lenucci M.S., Rescio L., Mita G., Caretto S. (2012). Durum wheat by-products as natural sources of valuable nutrients. Phytochem. Rev..

[3] Žilić S., Serpen A., Akıllıoğlu G., Janković M., Gökmen V. (2012). Distributions of phenolic compounds, yellow pigments and oxidative enzymes in wheat grains and their relation to antioxidant capacity of bran and debranned flour. J. Cereal Sci..

[4] Ciccoritti R., Carbone K., Bellato S., Pogna N., Sgrulletta D. (2013). Content and relative composition of some phytochemicals in diploid, tetraploid and hexaploid *Triticum* species with potential nutraceutical properties. J. Cereal Sci..

[5] Lampi A.M., Nurmi T., Ollilainen V., Piironen V. (2008). Tocopherols and tocotrienols in wheat genotypes in the HEALTHGRAIN diversity screen. J. Agric. Food Chem..

[6] Digesù A.M., Platani C., Cattivelli L., Mangini G., Blanco A. (2009). Genetic variability in yellow pigment components in cultivated and wild tetraploid wheats. J. Cereal Sci..

[7] Shewry P.R., Piironen V., Lampi A.M., Edelmann M., Kariluoto S., Nurmi T., Fernandez-Orozco R., Ravel C., Charmet G., Andersson A.A.M. (2010). The HEALTHGRAIN wheat diversity screen: Effects of genotype and environment on phytochemicals and dietary fiber components. J. Agric. Food Chem..

[8] Brenchley R., Spannagl M., Pfeifer M., Barker G.L.A., D’Amore R., Allen A.M., McKenzie N., Kramer M., Kerhornou A., Bolser D. (2012). Analysis of the bread wheat genome using whole-genome shotgun sequencing. Nature.

[9] Rawat N., Laddomada B., Gill B.S., Varshney R.K., Gupta P.K. (2013). Genomics of cereal-based functional foods. Cereal Genomics II.

[10] Rampino P., Mita G., Fasano P., Borrelli G.M., Aprile A., Dalessandro G., de Bellis L., Perrotta C. (2012). Novel durum wheat genes up-regulated in response to a combination of heat and drought stress. Plant Physiol. Biochem..

[11] Brandolini A., Castoldi P., Plizzari L., Hidalgo A. (2013). Phenolic acids composition, total polyphenols content and antioxidant activity of *Triticum monococcum*, *Triticum turgidum* and *Triticum aestivum*: A two-years evaluation. J. Cereal Sci..

[12] Li L., Shewry P.R., Ward J.L. (2008). Phenolic acids in wheat varieties in the HEALTHGRAIN diversity screen. J. Agric. Food Chem..

[13] Vitaglione P., Napolitano A., Fogliano V. (2008). Cereal dietary fibre: A natural functional ingredient to deliver phenolic compounds into the gut. Trends Food Sci. Technol..

[14] Lachman J., Hejtmánková K., Kotíková Z. (2013). Tocols and carotenoids of einkorn, emmer and spring wheat varieties: Selection for breeding and production. J. Cereal Sci..

[15] Liu R.H. (2007). Whole grain phytochemicals and health. J. Cereal. Sci..

[16] Medzhitov R. (2008). Origin and physiological roles of inflammation. Nature.

[17] Gianotti A., Danesi F., Verardo V., Serrazanetti D.I., Valli V., Russo A., Riciputi Y., Tossani N., Caboni M.F., Guerzoni M.E. (2011). Role of cereal type and processing in whole grain *in vivo* protection from oxidative stress. Front. Biosci..

[18] Harada A., Mukaida N., Matsushima K. (1996). Interleukin 8 as a novel target for intervention therapy in acute inflammatory diseases. Mol. Med. Today.

[19] Yoshimura T., Matsushima K., Tanaka S., Robinson E.A., Appella E., Oppenheim J.J., Leonard E.J. (1987). Purification of a human monocyte-derived neutrophil chemotactic factor that has peptide sequence similarity to other host defense cytokines. Proc. Natl. Acad. Sci. USA.

[20] Seitz M., Dewald B., Gerber N., Baggiolini M. (1991). Enhanced production of neutrophil-activating peptide-1/interleukin-8 in rheumatoid arthritis. J. Clin. Investig..

[21] Grimm M.C., Elsbury S.K., Pavli P., Doe W.F. (1996). Interleukin 8: Cells of origin in inflammatory bowel disease. Gut.

[22] Schroder J.M., Gregory H., Young J., Christophers E. (1992). Neutrophil activating proteins in psoriasis. J. Investig. Dermatol..

[23] Mahida Y.R., Ceska M., Effenberger F., Kurlak L., Lindley I., Hawkey C.J. (1992). Enhanced synthesis of neutrophil-activating peptide-1/interleukin-8 in active ulcerative colitis. Clin. Sci..

[24] Gibson P., Rosella O. (1995). Interleukin 8 secretion by colonic crypt cells* in vitro*: Response to injury suppressed by butyrate and enhanced in inflammatory bowel disease. Gut.

[25] Yomogida S., Hua J., Tsutsumi-Ishii Y., Sakamoto K., Nagaoka I. (2006). Effect of glucosamine on interleukin-8 production from human colonic epithelial cell line. Inflamm. Regen..

[26] Rieder F., Biancani P., Harnett K., Yerian L., Falk J.W. (2010). Inflammatory mediators in gastroesophageal reflux disease: Impact on esophageal motility, fibrosis, and carcinogenesis. Am. J. Physiol. Gastrointest. Liver Physiol..

[27] Drygiannakis I., Valatas V., Sfakianaki O., Bourikas L., Manousou P., Kambas K., Ritis K., Kolios G., Kouroumalis E. (2013). Proinflammatory cytokines induce crosstalk between colonic epithelial cells and subepithelial myofibroblasts: Implication in intestinal fibrosis. J. Crohns Colitis.

[28] Bahrami B., Macfarlane S., Macfarlane G.T. (2010). Induction of cytokine formation by human intestinal bacteria in gut epithelial cell lines. J. Appl. Microbiol..

[29] Hoffmann P., Sturm A., Stein J., Dignass A.U. (2011). Interferon-γ modulates intestinal epithelial cell function* in vitro* through a TGF-β-dependent mechanism. Regul. Pept..

[30] Macpherson A.J., Geuking M.B., Slack E., Hapfelmeier S., McCoy K.D. (2011). The habitat, double life, citizenship, and forgetfulness of IgA. Immunol. Rev..

[31] Whent M., Huang H., Xie Z., Lutterodt H., Yu L., Fuerst E.P., Craig F., Morris C.F., Yu L., Luthria D. (2012). Phytochemical composition, anti-inflammatory, and antiproliferative activity of whole wheat flour. J. Agric. Food Chem..

[32] Fernandez-Orozco R., Li L., Harflett C., Shewry P.R., Ward J.L. (2010). Effects of environment and genotype on phenolic acids in wheat in the HEALTHGRAIN diversity screen. J. Agric. Food Chem..

[33] Shewry P.R., Charmet G., Branlard G., Lafiandra D., Gergely S., Salgó A., Saulnier L., Bedȍ Z., Clare Mills E.N., Ward J.L. (2012). Developing new types of wheat with enhanced health benefits. Trends Food Sci. Technol..

[34] Dinelli G., Segura Carretero A., di Silvestro R., Marotti I., Fu S., Benedettelli S., Ghiselli L., Gutierrez A.F. (2009). Determination of phenolic compounds in modern and old varieties of durum wheat using liquid chromatography coupled with time-of-flight mass spectrometry. J. Chromatogr. A.

[35] Menga V., Fares C., Troccoli A., Cattivelli A., Baiano A. (2010). Effects of genotype, location and baking on the phenolic content and some antioxidant properties of cereal species. Int. J. Food Sci. Technol..

[36] Abdel-Aal E.M., Rabalski I. (2008). Bioactive compounds and their antioxidant capacity in selected primitive and modern wheat species. Open Agric. J..

[37] Chowers Y., Cahalon L., Lahav M., Schor H., Tal R., Bar-Meir S., Levite M. (2000). Somatostatin through its specific receptor inhibits spontaneous and TNFα and bacteria-induced IL-8 and IL-1β secretion from intestinal epithelial cells. J. Immunol..

[38] Dwinell M.B., Eckmann L., Leopard J.D., Varki N.M., Kagnoff M.F. (1999). Chemokine receptor expression by human intestinal epithelial cells. Gastroenterology.

[39] DeForge L.E., Fantone J.C., Kenney J.S., Remick D.G. (1992). Oxygen radical scavengers selectively inhibit interleukin 8 production in human whole blood. J. Clin. Investig..

[40] Massion P.P., Lindén A., Inoue H., Mathy M., Grattan K.M., Nadel J.A. (1996). Dimethyl sulfoxide decreases interleukin-8-mediated neutrophil recruitment in the airways. Am. J. Physiol..

[41] Hollebeeck S., Raas T., Piront N., Schneider Y.J., Toussaint O., Larondelle Y., During A. (2011). Dimethyl sulfoxide (DMSO) attenuates the inflammatory response in the* in vitro* intestinal Caco-2 cell model. Toxicol. Lett..

[42] Mateo Anson N., Aura A., Selinheimo E., Mattila I., Poutanen K., van den Berg R., Havenaar R., Bast A., Haenen G.R.M.M. (2011). Bioprocessing of wheat bran in whole wheat bread increases the bioavailability of phenolic acids in men and exerts antiinflammatory effects* ex vivo*. J. Nutr. Immunol..

[43] Lotito S.B., Zhang W.J., Yang C.S., Crozier A., Frei B. (2011). Metabolic conversion of dietary flavonoids alters their anti-inflammatory and antioxidant properties. Free Radic. Biol. Med..

[44] Kroon P.A., Clifford M.N., Crozier A., Day A.J., Donovan J.L., Manach C., Williamson G. (2004). How should we assess the effects of exposure to dietary polyphenols* in vitro*?. Am. J. Clin. Nutr..

[45] Manak C., Scalbert A., Morand C., Rémésy C., Jiménez L. (2004). Polyphenols: Food sources and bioavailability. Am. J. Clin. Nutr..

[46] Scalbert A., Williamson G. (2000). Dietary intake and bioavailability of polyphenols. J. Nutr..

[47] Yam M.L., Hafid S.R.A., Cheng H.M., Nesaretnam K. (2009). Tocotrienols suppress proinflammatory markers and cyclooxygenase-2 expression in RAW264.7 macrophages. Lipids.

[48] (1998). Mstatc-C Software, Version 2.6. Drinkwater N, Crop and Soil Science Department.

[49] Panfili G., Manzi P., Pizzoferrato L. (1994). High performance liquid chromatographic method for the simultaneous determination of tocopherols, carotenes and retinol and its geometric isomers in Italian cheese. Analyst.

[50] Fraser P.D., Pinto M.S.E., Holloway D.E., Bramley P.M. (2000). Application of high-performance liquid chromatography with photodiode array detection to the metabolic profiling of plant isoprenoids. Plant J..

[51] Re R., Pellegrini N., Proteggente A., Pannala A., Yang M., Rice-Evans C. (1999). Antioxidant activity applying an improved ABTS radical cation decolorization assay. Free Radic. Biol. Med..

[52] Minervini F., Fornelli F., Lucivero G., Romano C., Visconti A. (2005). T-2 toxin immunotoxicity on human B and T lymphoid cell lines. Toxicology.

